# The Emerging Roles of TERRA in Telomere Maintenance and Genome Stability

**DOI:** 10.3390/cells8030246

**Published:** 2019-03-15

**Authors:** Nicole Bettin, Claudio Oss Pegorar, Emilio Cusanelli

**Affiliations:** Laboratory of Cell Biology and Molecular Genetics, Department of Cellular, Computational and Integrative Biology - CIBIO, University of Trento, via Sommarive 9, 38123 Trento, Italy; nicole.bettin@gmail.com (N.B.); claudio.osspegorar@gmail.com (C.O.P.)

**Keywords:** TERRA, long noncoding RNA, telomeres, DNA damage response, genome stability, chromatin, human diseases

## Abstract

The finding that transcription occurs at chromosome ends has opened new fields of study on the roles of telomeric transcripts in chromosome end maintenance and genome stability. Indeed, the ends of chromosomes are required to be protected from activation of DNA damage response and DNA repair pathways. Chromosome end protection is achieved by the activity of specific proteins that associate with chromosome ends, forming telomeres. Telomeres need to be constantly maintained as they are in a heterochromatic state and fold into specific structures (T-loops), which may hamper DNA replication. In addition, in the absence of maintenance mechanisms, chromosome ends shorten at every cell division due to limitations in the DNA replication machinery, which is unable to fully replicate the extremities of chromosomes. Altered telomere structure or critically short chromosome ends generate dysfunctional telomeres, ultimately leading to replicative senescence or chromosome instability. Telomere biology is thus implicated in multiple human diseases, including cancer. Emerging evidence indicates that a class of long noncoding RNAs transcribed at telomeres, known as TERRA for “TElomeric Repeat-containing RNA,” actively participates in the mechanisms regulating telomere maintenance and chromosome end protection. However, the molecular details of TERRA activities remain to be elucidated. In this review, we discuss recent findings on the emerging roles of TERRA in telomere maintenance and genome stability and their implications in human diseases.

## 1. Introduction

Telomeres are nucleoprotein structures assembled at the end of eukaryotic chromosomes, protecting them from degradation, fusion, and erroneous recombination events [1,2,3,4]. Chromosome ends consist of tandem arrays of a short DNA sequence (TTAGGG_n_ in vertebrates), or telomeric repeat, which terminate in a single-stranded G-rich 3′ overhang [5,6,7,8]. Telomere length varies considerably among organisms: *Mus musculus* has very long telomeres (20 to 50 kb) as compared to *Homo sapiens* telomeres (5 to 15 kb) and *S. cerevisiae* or *S. pombe* telomeres (~300 bp) [5]. Electron microscopy and super-resolution fluorescence microscopy studies revealed that telomeric DNA can fold into higher-order structures in which the single-stranded overhang invades the homologous double-stranded region, forming a telomeric loop (T-loop) [9,10]. In addition, the G-rich telomeric repeats can fold into G-quadruplex structures that are composed of square planar alignments of four guanine rings (G-quartet), stabilized by hydrogen bonds between neighboring guanines [11,12]. Telomeric DNA structures have important implications in telomere biology [13,14,15].

Telomeric repeats are bound by a set of telomere-binding proteins that mediate telomere functions and regulate telomere maintenance [16]. In mammals, telomere binding proteins form the so-called “shelterin” complex. In human cells, the shelterin complex consists of six proteins that are recruited to telomeres through the direct binding of the shelterin subunits TRF1 and TRF2 to the double-stranded telomeric repeats [16,17,18,19]. The shelterin components POT1 and TPP1 interact as a heterodimer with the single-stranded 3′ overhang, while TIN2 links the POT1/TPP1 heterodimer to TRF1 and TRF2, and stabilizes the association of TRF1 and TRF2 with chromosome ends [20]. The shelterin subunit Rap1 interacts with TRF2, increasing its specificity of binding for telomeric DNA and regulating its localization at chromosome ends [21,22].

A key function of telomeres is to enable the cell to discriminate the natural ends of chromosomes from harmful double-strand breaks (DSBs) [16,17]. This function is mainly mediated by TRF2 and POT1, which prevent chromosome ends from activating DNA damage signaling and DSB repair pathways [16,23]. TRF2 is required for T-loop formation and maintenance [10]. The T-loop structure can sequester the 3′ end of chromosomes, thereby preventing its recognition by the DNA damage response (DDR) machinery [24,25]. In addition, TRF2 represses the ATM kinase-mediated DNA damage response and the non-homologous end joining (NHEJ) repair pathway by regulating the formation of the 3′ overhang at the leading-end telomeres [26]. The POT1-TPP1 heterodimer plays a key role in repressing the ATR kinase-mediated DNA damage response, most likely by competing with the replication protein A (RPA) for the binding to the 3′ overhang [23]. TRF1 and TRF2 recruit the Bloom syndrome protein (BLM) helicase and the regulator of telomere elongation helicase 1 (RTEL1), respectively, in order to unwind G-quadruplexes and unfold T-loop structures, that would otherwise pose an obstacle to the replication of telomeric DNA [27,28,29]. Helicases activity enables the progression of the replication fork through telomeric DNA, preventing replication fork stalling and consequent activation of DNA damage signaling [16,30,31]. 

Nevertheless, the DNA replication machinery is unable to fully replicate the extremities of a linear double-stranded DNA molecule [32]. As a consequence, in the absence of maintenance mechanisms, chromosome ends shorten at every cell division creating the so-called “end replication problem” [33]. Continuous loss of telomeric repeats can result in decreased amount of shelterin proteins associated to chromosome ends [34,35]. Short telomeres eventually become dysfunctional and are recognized as DNA damaged sites [36]. Sustained activation of the DNA damage response at chromosome ends ultimately triggers replicative senescence through the activity of p53 and Rb signaling [37,38]. In order to counteract telomere shortening, most eukaryotic cells express a dedicated reverse transcriptase enzyme called telomerase, which adds telomeric repeats to the 3′ end of chromosomes by reverse transcription of the template region of its associated RNA moiety [39,40]. The shelterin complex is required for telomerase recruitment and activity at telomeres [40,41,42,43,44]. While telomerase is expressed in proliferating cells, such as germ cells and stem cells, telomerase is not expressed in human somatic cells, which enter replicative senescence upon a defined number of cell divisions [33]. Replicative senescence acts as a tumor suppressor mechanism by limiting the proliferative capacity of cells and inhibiting cellular transformation [45]. Accordingly, 90% of human cancers reactivate telomerase in order to maintain telomere length and attain unlimited proliferative capacity [46]. The remaining 10% of cancers maintain their telomeres in the absence of telomerase [47]. In these cells, telomere length homeostasis is achieved by homologous recombination-mediated mechanisms known as alternative lengthening of telomeres, or “ALT” [48]. Thus, telomere length homeostasis acts as a genetic clock that regulates cellular life span and is inextricably linked to tumorigenesis [45]. 

Telomeres are enriched in heterochromatic marks, including trimethylated lysine 20 of histone H4 (H4K20me3) and lysine 9 of histone H3 (H3K9me3), heterochromatin protein 1 (HP1) proteins and methylated cytosines [49,50]. The epigenetic signature of telomeric chromatin has been proposed to regulate telomerase activity and ALT mechanisms [51,52,53]. In particular, loss of histone methyltransferases results in over-elongated telomeres in mouse [53]. Furthermore, compromised telomeric heterochromatin formation may provide a recombination-permissive environment that promotes ALT development [48,51,52,54,55,56]. Interestingly, telomeres are transcribed, giving rise to a class of long noncoding RNA called telomeric repeat-containing RNAs, or TERRA [57,58]. A growing body of evidence indicates that TERRA actively participates in the mechanisms regulating telomere function and telomere homeostasis [59]. Several studies point to TERRA as a scaffold molecule promoting the recruitment of proteins and enzymatic activities at chromosome ends. In this review, we discuss recent findings on the emerging roles of TERRA in telomere maintenance and genome stability and their implications in human diseases.

## 2. TERRA and Chromatin Regulation

TERRA molecules are transcribed from the subtelomeric regions towards chromosome ends by RNA Pol II using the telomeric C-rich strand as the template [59]. For this reason, TERRA transcripts consist of subtelomeric-derived sequences at their 5′ end and terminate with tandem arrays of G-rich telomeric sequences. At Northern blot analyses, TERRA molecules resolve as highly heterogeneous transcripts ranging from 100 nt to 9 kb in mammalian cells [57,58]. In human cells, most of TERRA is 7-methylguanosine (m^7^G) capped at its 5′-end, while only 7% of the total TERRA is polyadenylated at its 3′-end [58,60]. Polyadenilated TERRA transcripts exhibit a longer half-life (8 h) as compared to nonpolyadenylated TERRA (3 h) [57,61]. The mechanism of TERRA polyadenylation remains to be elucidated, as a canonical polyadenylation signal is not present at telomeres. In human cells, TERRA is transcribed from CpG islands located within a subset of subtelomeric regions, in the proximity of the telomeric repeat tract [62]. A second class of TERRA promoters were identified in HeLa human cervical carcinoma cells at about 5–10 kb from telomeric tract of 10 distinct chromosome ends [63]. Interestingly, both classes of TERRA promoter regions contain binding motifs for the chromatin organizing factor CTCF (CCCTC-binding factor), which acts as a key regulator of TERRA expression (discussed below) [63,64]. TERRA expression has been shown to be cell-cycle-dependent, with TERRA levels peaking in G1 and progressively decreasing during S phase in human cells [61].

**Figure 1:**
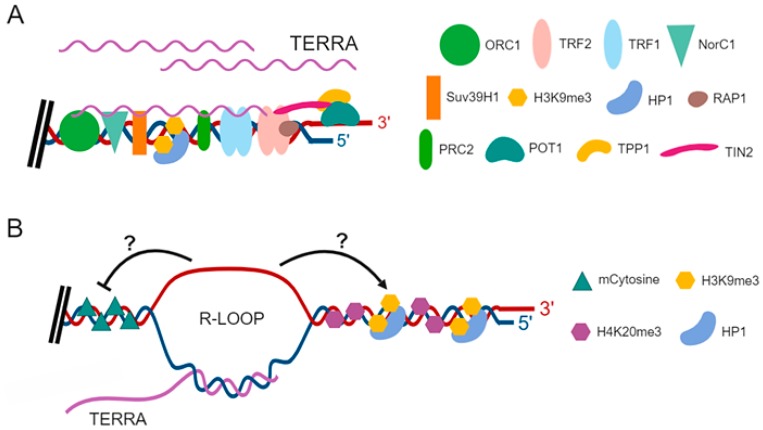
Proposed roles of TERRA in chromatin regulation at telomeres. (**A**) TERRA interacts with various proteins at telomeres including TRF2, Suv39h1, and origin replication complex 1 (ORC1). TERRA transcripts can act as scaffold molecules to promote the recruitment of proteins, histone modifying enzymes and chromatin remodeling complexes to chromosome ends. (**B**) TERRA forms DNA-RNA hybrid structures (R-loops) at telomeres. Telomeric R-loops can influence heterochromatin formation at telomeres by inhibiting DNA methylation or promoting the recruitment of HP1 proteins at chromosome ends.

Notably, since telomeric repeat-like sequences are present at internal chromosomal loci, also known as ITSs for Interstitial Telomeric Sequences, in a variety of organisms [65], telomeric repeat-containing RNAs may in principle be also expressed from internal chromosomal regions. However, telomeres consist for their most part of tandem arrays of perfect telomeric repeats, while ITSs are generally composed of telomeric repeats interspaced with degenerate repeats [66,67]. By using a specific RT protocol to measure the length of the telomeric repeat tract of TERRA, it was observed that the vast majority of TERRA transcripts terminates at its 3′ end with perfect UUAGGG repeats ranging in length from 100 bases to 400 bases, in HeLa cells and human lung fibroblasts (HLF) [61]. These findings suggest that, if expressed, TERRA transcribed from ITSs can represent a negligible fraction of the total TERRA population. In addition, a variety of studies have used telomeric repeat-specific RT primers and subtelomere-specific PCR and qPCR primers to show that TERRA is expressed from multiple chromosome ends in human cells [62,63,68,69,70]. In line with this evidence, TERRA transcription is regulated by the heterochromatic state of telomeres and subtelomeric regions. Indeed, methylation of telomeric DNA as well as H3K9 and H4K20 trimethylation repress TERRA expression [57,62,69,71]; instead histone acetylation positively associates with TERRA transcription [72]. The heterochromatic state of telomeres regulates TERRA expression also in yeast [73]. Interestingly, emerging evidence indicates that TERRA transcription plays a key role in the regulation of heterochromatin formation at telomeres (Figure 1). In mammalian cells, TERRA transcripts interact with heterochromatic marks, including H3K9me3 and HP1 proteins [74], the histone methyltransferase Suv39h1 [63] and with chromatin remodeling complexes, such as NoRC (nucleolar remodeling complex) [75], MORF4L2, a component of the NuA histone acetyltransferase complex, and ARID1A, a component of the BAF-type SWI/SNF nucleosome remodeling complex [76]. In addition, recent evidence indicates that TERRA transcripts associate with the histone methyl transferase Polycomb Repressive Complex 2 (PRC2), through a direct interaction with the PRC2 components EZH2 and SUZ12 [77,78,79]. In the osteosarcoma cell line U2OS, an ALT human cancer cell line expressing high levels of TERRA, the TERRA-PRC2 interaction promotes deposition of H3K9me3, H4K20me3, H3K27me3, and HP1 protein to chromosome ends [77] (Figure 1A). Notably, evidence indicates that telomeres in ALT cells are in a less compact state than in telomerase-positive cells [48,54,56]. These findings may explain at least in part the induced levels of TERRA in ALT cells with respect to telomerase-positive cancer cells [54,80]. Nevertheless, it has been recently shown that U2OS cells have heterochromatic levels of H3K9me3 [64,66]. The high levels of TERRA in U2OS cells may thus contribute to the epigenetic signature of these cells. Interaction of TERRA with PRC2 depends, at least in part, on the structure of TERRA transcripts. Indeed, the G-rich 3′ end of human TERRA forms G-quadruplex structures both in vivo and in vitro [81,82,83]. The PRC2 complex preferentially binds single-stranded RNAs containing G-rich repetitions and has high affinity for G-quadruplex structures [79]. It has been shown that TERRA G-quadruplex structure acts as a binding target for other telomere-binding proteins, such as the translocated in liposarcoma/fused in sarcoma (TLS/FUS) protein [84] and TRF2 [74,85]. Interestingly, recent in vitro experiments have shown that TLS/FUS forms a ternary complex with the human telomere G-quadruplex DNA and the G-quadruplex TERRA structure [86]. These interactions can promote trimethylation of histone H4 at lysine 20 (H4K20me3 modification) at telomeres through the activity of the histone methyltransferase Suv4-20h2 that associates with TLS/FUS [84]. 

Interaction of TERRA with the shelterin components TRF1 and TRF2 may anchor TERRA transcripts to chromosome ends, sustaining the enzymatic activities of TERRA binding factors at telomeres. In support of this hypothesis, expression of a TRF2 mutant protein (TRFΔB) impairs TERRA localization at telomeres of U2OS cells [74]. However, TRF1 and TRF2 also bind extratelomeric sites, in particular ITSs [87,88]. Thus TERRA/TRF proteins interaction may not be limited to chromosome ends and may be involved in the recruitment of TERRA transcripts to extratelomeric sites. Indeed, RNA FISH analyses have shown that only a subset of TERRA molecules accumulates at telomeres [89] and live cell imaging studies revealed that TERRA transcripts only transiently localize at chromosome ends, in humans as in yeast [90,91,92]. These findings suggested that TERRA molecules may also bind to extratelomeric regions of chromosomes. Recently, Chu et al. developed a CHIRT protocol from a combination of the ChiRP (CHromatin isolation by RNA Purification) and CHART (Capture Hybridization Analysis of RNA Targets) methods to investigate the genomic binding sites of TERRA transcripts in mouse embryonic stem cells (mESCs) [78]. Interestingly, it was observed that most TERRA binding sites map at extratelomeric loci, particularly at intergenic regions and introns. These findings indicate that TERRA binds both at telomeres and within or near genes. In the same study, antisense oligonucleotides (ASO) containing locked nucleic acids (LNA) were designed to promote RNAse H-mediated degradation of TERRA transcripts. Notably, TERRA depletion resulted in dysregulation of hundreds of genes containing TERRA binding sites as detected by transcriptomic analyses in mESCs [78]. Taken together, these results suggest that TERRA transcripts regulate the epigenetic signature of chromatin and gene expression at subtelomeres as well as extratelomeric loci. It will be important to further study whether TERRA transcripts can exert these extratelomeric functions also in human cells and mouse embryonic fibroblasts.

## 3. TERRA and Telomere Maintenance

Downregulation of TERRA transcripts achieved by siRNA [74] or ASO-LNA [78,93] results in the activation of DNA damage responses at chromosome ends and in the consequent formation of “Telomere dysfunction-induced foci” (TIFs). Furthermore, unscheduled association of TERRA at chromosome ends results in TIFs formation and chromosome abnormalities [58,94], suggesting that a tight regulation of TERRA expression and localization is required to maintain telomere and chromosome stability [95].

The role of TERRA in telomere maintenance was recently studied by Montero et al., who used CRISPR/Cas9 to remove the TERRA locus from telomere 20q in U2OS cells. The TERRA20q-KO clones showed a marked decrease in the overall TERRA population, suggesting that TERRA transcripts expressed from this telomere represent a substantial fraction of the total TERRA population in U2OS cells [96]. Furthermore, the TERRA20q-KO clones showed increased DNA damage at chromosome ends, telomere fusions, and decreased telomere length. These findings suggest that TERRA-20q transcripts act *in trans* by participating in telomere protection at multiple chromosome ends in U2OS cells [96]. Similar results were previously obtained in mouse embryonic fibroblasts (MEF) by the same group, reporting that partial depletion of TERRA expressed from the single telomere 18q resulted in activation of DNA damage response and telomere dysfunction at multiple chromosome ends [93]. Worthy of note, also in this study it was observed that TERRA transcribed from a single telomere (telomere 18q) represents the primary source of TERRA, acting *in trans* on multiple chromosome ends [93]. Conversely, other studies have reported that TERRA transcripts are expressed from multiple telomeres in ALT cancer cells [54,62,64], and it has been shown that mouse ES cells express TERRA mainly from the subtelomeric pseudoautosomal regions (PAR) of the two sex chromosomes X and Y [97]. In some occasions, the different results may be due to the different cell lines used in the various studies (IMRB [54] vs. U2OS [96] ALT cell lines and MEF [93] vs. mESCs [97]). Distinct telomeres may contribute to the expression of total TERRA in a cell line dependent manner. In addition, ALT cancer cells display a high rate of rearrangements and telomere heterogeneity, which can impact on TERRA expression and regulation. Cells cultured for different number of passages may display variations in TERRA expression. Not least, the specific methods and protocols used to analyze TERRA expression may contribute to the different results obtained in different studies [98]. To date, all studies on TERRA using telomerase-positive human cancer cells have reported TERRA expression from multiple chromosome ends [62,63,69,71].

**Figure 2:**
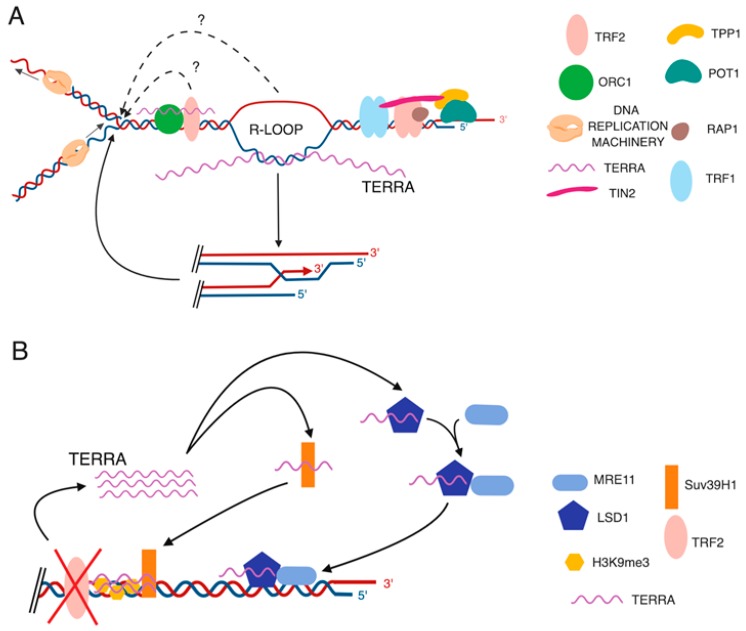
Proposed TERRA functions in telomere maintenance. (**A**) TERRA transcripts may facilitate DNA replication at telomeres by promoting the recruitment of the origin replication complex 1 (ORC1) at chromosome ends or through the formation of DNA-RNA hybrids (R-loops) at chromosome ends. Telomeric R-loops promote homologous recombination among telomeres, which protects replication forks from collapsing and becoming dysfunctional during replication stress. (**B**) TERRA sustains DNA damage response at dysfunctional telomeres. Depletion of TRF2 results in increased TERRA levels. TERRA transcripts promote the recruitment of LSD1-Mre11 complex at chromosome ends, which can fuel DNA damage response at dysfunctional telomeres. TERRA also interacts with Suv39h1 histone methyltransferase. In TRF2-depleted cells, TERRA-Suv39h1 interaction promotes H3K9me3 accumulation at dysfunctional telomeres, sustaining the DNA damage response.

The expression of TERRA is regulated by the chromatin organizing factor CTCF (CCCTC-binding factor) and the cohesin Rad21 (radiation-sensitive 21), which associate with the CpG island-containing TERRA promoters present within multiple human subtelomeres [63,64]. CTCF has been implicated in numerous processes including nucleosome positioning [99] and transcription regulation [100]. Rad21 is a component of the cohesin complex, which mediates sister chromatids cohesion by forming ring-shaped structures holding sister DNA molecules together [101]. CTCF and cohesion colocalize on chromosomes and help regulate genome folding, thereby impacting transcription, genome replication and genome integrity [102,103]. In U2OS cells and HCT116 human colon cancer cells, CTCF and Rad 21 promote the recruitment of RNA Pol II to the TERRA promoter region and shRNA-mediated depletion of Rad21 or CTCF results in loss of RNA Pol II binding at the TERRA promoters and consequent decrease in TERRA levels [64]. Surprisingly, depletion of Rad21 or CTCF resulted in reduced binding of TRF1 and TRF2 to telomeres without influencing the total levels of the two proteins. As discussed by Deng et al. in their study, a possible explanation for these findings is that CTCF and Rad21 influence the RNA pol II binding and local histone modifications that are required for proper maintenance of telomeric chromatin and its association with shelterin. Notably, CTCF and Rad21 depletion lead to an increase in activation of DNA damage responses at chromosome ends and TIFs formation [64]. Since depletion of TERRA using siRNAs was previously shown to result in TIFs formation in U2OS cells [74], CTCF and Rad21 may participate in chromosome ends protection by recruiting RNA pol II to subtelomeres and promoting TERRA expression. Interestingly, the same laboratory recently used genome editing tools to specifically delete subtelomeric CTCF-binding sites present within subtelomere 17p [104]. HCT116 cells lacking subtelomeric CTCF-binding sites exhibited a marked decrease in TERRA expression from the engineered telomere and a significant decrease in the recruitment of histone H3K4me3 at the same chromosome end. Notably, the HCT116 mutant cells were sensitive to replicative stress and the TERRA-lacking telomere exhibited impaired DNA replication, which resulted in formation of ultra-fine anaphase bridges during replicative stress conditions [104]. Telomeres represent genomic regions notoriously difficult to be replicated due to their repetitive sequences and structure [31,105]. This study from Beishline et al. suggests that TERRA transcripts facilitate DNA replication *in cis*, at their telomere of origin [104]. In line with this evidence, TERRA transcripts have been shown to interact with origin replication complexes (ORCs) [74,78]. In addition, TERRA can directly bind both TRF2 and ORC1 [74]. The formation of this ternary complex may facilitate DNA replication at telomeres. TERRA transcripts may also assist telomeric DNA replication by regulating heterochromatin formation [106], while the mere act of transcription of TERRA may positively impact on the replication timing of telomeres [107,108,109] (Figure 2A). Indeed, it has been observed that transcription during S phase associates with early firing of the nearby origin of replication in yeast and human cells, possibly due to the presence of a more accessible chromatin structure [108]. In addition, RNA polymerase has been shown to promote the sliding of the replicative helicase MCM2-7 complexes favoring initiation of DNA replication at sites distant from ARS elements in yeast [109]. Whether these mechanisms influence the replication of telomeric DNA remains to be determined. 

Recently, TERRA transcripts expressed from subtelomere 15q were tagged using a short MS2 sequence integrated at the subtelomere 15q TERRA locus in the human stomach cancer cell line AGS [90]. The MS2-tagged TERRA transcripts were visualized by fluorescence microscopy by co-expressing a GFP-fused MS2 RNA binding protein (MS2-GFP). This approach enabled the following of single-telomere TERRA transcripts localization in living cells by fluorescence microscopy revealing that TERRA molecules transiently localize at chromosome ends [90]. It remains to be determined whether MS2-tagged TERRA transcripts preferentially associate with their telomere of origin, as previously observed in yeast [91]. Importantly, depletion of MS2-tagged TERRA transcripts via ASO-LNA resulted in the activation of DNA damage responses predominantly at extratelomeric regions and at a single telomere signal, as detected by immunofluorescence experiments [90]. These findings suggest that TERRA transcribed from a single telomere can impact on DNA damage responses at telomeres and extratelomeric sites.

## 4. Proposed TERRA Mechanisms of Action in Telomere Maintenance

How can TERRA participate in telomere maintenance and chromosome ends protection from DNA damage response activation? In addition to facilitating completion of telomeric DNA replication, TERRA transcripts may prevent activation of DNA damage responses at telomeres by promoting the proper assembly of telomere binding proteins at chromosome ends or telomere capping. Indeed, in vitro evidence has suggested that a TERRA-hnRNPA1 complex favors the association of POT1 to the telomere by promoting the displacement of RPA, which competes with POT1 for the binding to the telomeric single-strand DNA [110]. TERRA may also indirectly act on DNA damage response pathways by regulating gene expression [78]. Importantly, both human and yeast TERRA transcripts have been shown to form DNA-RNA hybrid structures at telomeres, known as R-loops [111,112,113,114,115]. Interestingly, in the absence of telomerase, TERRA R-loops accumulate at critically short telomeres and their metabolism is regulated during cell cycle in yeast [116]. In mammalian cells, elevated levels of telomeric R-loops are detected in telomerase-negative ALT cancer cells [114], which express higher levels of TERRA as compared to telomerase-positive cancer cells [54,80]. R-loop formation at telomeres can regulate telomere maintenance and influence genome stability by various mechanisms, including chromatin regulation [117,118,119], priming the initiation of DNA replication [120], or promoting homologous recombination among telomeres [114,120,121] (Figure 1B and Figure 2B). Notably, homologous recombination can protect DNA replication forks from collapsing and becoming dysfunctional [122]. Thus, TERRA may sustain telomeric DNA replication through R-loop formation (Figure 2A). Emerging evidence indicates that telomeric R-loops may play a role in telomere maintenance of ICF (immunodeficiency, centromeric instability and facial anomalies) syndrome cells [123]. The ICF syndrome is a rare autosomal recessive disease caused by mutations in the DNA methyltransferase 3b (DNMT3b) gene, which result in spontaneous genomic instability and immunodeficiency [124]. ICF cells have short telomeres, express high TERRA levels, and exhibit elevated rates of telomere instability [125,126,127,128]. Interestingly, ectopic expression of RNase H1, which degrades the RNA component of DNA-RNA hybrids, resolving the R-loops, significantly reduced the DNA damage at chromosome ends of ICF cells [123]. These findings suggest that accumulation of R-loops can sustain telomere instability in ICF cells. The regulation and functions of DNA-RNA hybrids at telomeres have been recently reviewed in detail [129]. 

Notably, TERRA expression may act as a double-edged sword in the regulation of the DNA damage response (DDR). Indeed, TERRA transcription is induced during telomere dysfunction upon depletion of TRF2 [63,130]. In this setting, TERRA can contribute to the DDR at telomeres by promoting the recruitment of the TERRA-interacting factors lysine-specific demethylase (LSD1) and histone methyltransferase Suv39h1 to chromosome ends (Figure 2B). LSD1 can promote the recruitment of the Mre11/Rad50/NBS1 (MRN) complex, an early sensor of DNA damage, to telomeres, while Suv39h1 activity can sustain activation of ATM, an apical kinase of the DDR, by promoting H3K9me3 at telomeres [63,130,131]. Furthermore, depletion of components of the nonsense-mediated mRNA decay (NMD) pathway [58] or TERRA-interacting members of the heterogeneous nuclear ribonucleoprotein family (hnRNPs) [94], results in unscheduled localization of TERRA to chromosome ends, without influencing TERRA expression levels. This localization of TERRA is associated with TIFs formation and chromosome abnormalities [58,94]. Interestingly, increased localization of TERRA to chromosome ends upon depletion of the up-frameshift 1 (UPF1) member of the NMD pathway also associates with impaired telomere leading-strand replication in HeLa cervical cancer cells [132]. Thus, not only loss of TERRA expression can impair telomeric DNA replication *in cis*, at the TERRA-lacking telomeres [104], but also sustained localization of TERRA to chromosome ends can associate with compromised telomere DNA replication. In this regard, a better understanding of the dynamics of TERRA molecules, for example by characterizing whether TERRA transcripts act only *in cis*, at the transcribing telomere, or also *in trans* by relocating to other chromosome ends can help to define the functions of TERRA. The observation that a TERRA-depleted telomere manifests sister-telomere loss and ultra-fine anaphase bridges only during replicative stress [104] could in principle be explained by an impaired localization of TERRA transcripts during replication stress. In this scenario, loss of TERRA from an engineered telomere may be compensated by the relocation of TERRA molecules from other telomeres to the TERRA-lacking telomere during normal culturing conditions. Impaired localization of TERRA under replication stress would alter such fail-safe mechanism. However, the dynamics of TERRA during replicative stress conditions remain to be defined. Overall, the above-mentioned findings further indicate that TERRA expression and localization must be tightly regulated in cells: while on one hand TERRA transcripts can help to protect telomeres from activation of the DDR, on the other hand the induction of TERRA expression or the altered localization of TERRA transcripts can negatively impact telomeric DNA replication or fuel the DDR at chromosome ends during telomere dysfunction.

## 5. TERRA under Stress

An interplay between TERRA expression, the DNA damage response, and cellular stress is emerging. Indeed, early studies reported that TERRA expression is induced in murine embryonic fibroblasts (MEF) cultured under heat shock stress conditions [57]. Recent evidence has now revealed that TERRA transcription during heat shock is directly induced by the transcription factor heat shock factor 1 (HSF1), which binds to subtelomeric regions during stress conditions in human cells [133]. Accordingly, the induction of TERRA during heat shock is prevented in HSF1 knock out cells. Interestingly, heat shock of HSF1-KO cells results in increased DNA damage at telomeres and TIF formation as compared to WT cells cultured in the same conditions [133]. These findings suggest that TERRA induction may help to preserve telomere integrity during cellular stress.

In line with this view, TERRA expression is induced upon treatment of HCT116 cells with the chemotherapeutic agent etoposide or upon culturing these cells in serum-free medium [134]. Notably, induction of TERRA during these stress conditions is dependent on the p53 transcription factor, which binds the subtelomeric regions on a variety of chromosomes [134]. Induction of TERRA during stress was impeded in p53 knock-out cells, which also showed increased DNA damage at telomeres and TIFs formation. Furthermore, deletion of a p53 binding site within subtelomere 18q impeded the induction of telomere 18q TERRA and the nearby subtelomeric gene PARD6G upon etoposide treatment in HCT116 cells. Impaired expression of TERRA and PARD6G gene resulted in increased DNA damage at telomere 18q upon etoposide treatment. These findings suggest that TERRA induction participates in a p53-mediated protection response of human telomeres upon replicative stress [134]. It will be interesting to verify whether this mechanism also occurs under other stress conditions or physiological conditions.

In addition, recent evidence indicates that TERRA expression oscillates during yeast cell growth and TERRA levels are induced when yeast cells undergo diauxic shift, a lag phase during which cells switch their metabolism from fermentation to oxidative respiration, due to the limiting amount of glucose in the medium [135]. Interestingly, in contrast with the nuclear localization of TERRA during the logarithmic phase of growth, during diauxic shift TERRA transcripts localize to the cytoplasm [135]. These findings suggest that TERRA may play a role in the response to oxidative stress resulting from the oxidative respiration metabolism of diauxic shift cells. In this regard, it has been recently shown that TERRA transcripts localize to telomeres in human muscle tissues and TERRA expression is upregulated by the antioxidant transcription factor NRF1 during endurance exercise in human myofibers [136]. In particular, Diman et al. showed that NRF1 interacts with CpG island-containing TERRA promoters on multiple subtelomeres in LB37 non-small cell lung carcinoma and Huh-7 hepatocarcinoma human cell lines. NRF1 binding to subtelomeric DNA was positively correlated with TERRA expression levels. The authors found that TERRA expression was induced in muscle biopsies obtained from healthy volunteers who submitted to cycling endurance exercise. TERRA induction associated with activation of peroxisome proliferator-activated receptor ɣ coactivator 1α (PGC-1a), which is a regulator of energy metabolism upon caloric restriction and endurance exercise, acting as coactivator of transcription factors, including NRF1. The authors further dissect the mechanism of TERRA expression regulation in human myotubes showing that TERRA expression is regulated by NRF1 and PGC-1a, the latter being activated by the adenosine 5′-monophosphate (AMP)-activated protein kinase (AMPK), which is positively regulated during exercise. Downregulation of NRF1 induces TIFs formation in Huh-7 cells, suggesting that the NRF1 antioxidant factor participates in telomere maintenance mechanisms. Telomeric DNA is particularly sensitive to oxidative damage due to its high content in guanines, which can be oxidized to 8-oxo guanines (8-oxoG) [137]. This base modification can impair telomere structure and function [138]. The findings of Diman et al. suggest that TERRA transcripts may participate in an antioxidant response triggered by telomeres in skeletal muscles during endurance exercise. A better understanding of the spatiotemporal dynamics of TERRA during cellular stress will help to define possible novel functions of TERRA during stress response.

## 6. Concluding Remarks

As discussed in this review, clear evidence shows that TERRA is an important player in telomere maintenance and genome stability. In addition, TERRA is a direct target of important tumor suppressor genes, such as p53 [134] and Rb [139], further indicating that TERRA transcripts can be crucially involved in tumorigenesis. In line with this view, it has been recently shown that TERRA is negatively regulated by the SNAIL transcription factor, which plays a key role in the epithelial-to-mesenchymal transition (EMT) of tumor cells [140]. Interestingly, SNAIL-mediated repression of TERRA influences the EMT of murine mammary gland epithelia (NMuMG) cells [140]. As novel functions of TERRA and different biological processes in which it participates are being proposed and discovered, understanding the specific mechanisms of action of TERRA may become more challenging. Since TERRA expression is highly conserved through evolution [57], the use of model organisms such as mice [141] or zebrafish [142] may become a powerful tool to study TERRA and to understand its function and mechanisms of action. In doing so, it should be considered that the dynamics and functions of TERRA may not be conserved among the different models. For example, TERRA interacts with telomerase in yeast as well as human and mouse cells [78,91,143]. This interaction may involve a direct pairing of the G-rich 3′ end of TERRA with the template region of telomerase RNA [57,143], which could in principle inhibit telomerase activity. Supporting this model, TERRA-mimicking oligonucleotides have been shown to inhibit human and mouse telomerase in vitro [57,143] and TERRA depletion through TERRA-ASO associates with increased telomerase activity in mESCs [78]. However, TERRA has been proposed to positively regulate telomerase activity at chromosome ends in budding yeast *S. cerevisiae* [91] and fission yeast *S. pombe* [144]. Furthermore, induced expression of TERRA from a single engineered telomere containing a TERRA inducible promoter as well as the high levels of TERRA in DNA methyltransferases-deficient HCT116 cells do not interfere with the activity of telomerase or telomere length homeostasis [69]. While the molecular details of the role of TERRA in the regulation of telomerase need to be elucidated, these findings suggest that TERRA functions may not be conserved among different organisms.

Studies of TERRA localization may pave the way for the understanding of TERRA functions. Indeed, early studies on TERRA revealed that TERRA transcripts localize to the X and Y chromosomes in mouse embryonic stem cells (mESCs) and human embryonic stem cells [57,145]. Interestingly, while TERRA colocalize with both sex chromosomes in undifferentiated cells, it only localizes to the heterochromatic sex chromosome during differentiation [57,145]. Recent investigations have provided further insights on this intriguing observation and it has been shown that the subtelomeric pseudoautosomal regions (PAR) of the two sex chromosomes, X and Y, are transcribed into telomeric repeat-containing RNAs, which were named “PAR-TERRA” in mESCs [97]. At Northern blot analyses, PAR-TERRA resolves as a heterogeneous population of transcripts recapitulating the size distribution of TERRA transcripts. In addition, RNA FISH experiments revealed overlapping subnuclear localization between TERRA and PAR-TERRA in mESCs. Notably, PAR-TERRA accounts for the majority of TERRA transcripts in mESCs. Intriguingly, PAR-TERRA has been shown to promote somatic homologous X-chromosome pairing, which is required for proper X chromosome inactivation (XCI) [97]. Thus, studying the dynamics of TERRA molecules may help to unveil unexpected functions and regulation. It will be interesting to further investigate the expression and function of PAR-TERRA in human ES cells.

It may be important to consider that TERRA is not the only transcript expressed from telomeres. In fission yeast, telomeres are transcribed into TERRA, its antisense RNA “ARIA” (mainly consisting of C-rich telomeric repeats), and two complementary subtelomeric long noncoding RNAs called “ARRET” and “α-ARRET” [146,147,148]. These transcripts are not detected in mammalian cells, while ARRET is expressed in budding yeast [149]. However, telomeric transcripts are negatively regulated by the fission yeast telomere-binding proteins Taz1 and Rap1, suggesting that the expression of telomeric RNAs may be dependent on the state of telomeres (functional vs. dysfunctional, short vs. long) even in other organisms. In this regard, C-rich telomeric transcripts have recently been described in mammalian cells [150]. Interestingly, Rossiello et al. reported that dysfunctional telomeres induce transcription of telomeric DNA damage response RNA (tDDRNA) [150]. Depletion of tDDRNA by ASO-LNA results in inhibition of the DNA damage response at telomeres, indicating that tDDRNAs are important regulators of DDR at chromosome ends. It will thus be of interest in future studies to investigate the potential interplay between TERRA and tDDRNAs in telomere function and genome stability.
